# Comparison of endoscopic evacuation, stereotactic aspiration and craniotomy for the treatment of supratentorial hypertensive intracerebral haemorrhage: study protocol for a randomised controlled trial

**DOI:** 10.1186/s13063-017-2041-1

**Published:** 2017-06-28

**Authors:** Xinghua Xu, Yi Zheng, Xiaolei Chen, Fangye Li, Huaping Zhang, Xin Ge

**Affiliations:** 1 Department of Neurosurgery, Chinese PLA General Hospital, Fuxing Road 28, Beijing, 100853 China; 2 Department of Dermatology, Beijing Chaoyang Hospital, Capital Medical University, Gongren Tiyuchang Nanlu 8, Beijing, 100020 China; 3 Department of Neurosurgery, Jingzhou Central Hospital, Jingzhong Road 60, Hubei, 424000 China; 4 Department of Neurosurgery, Jinzhou Central Hospital, Shanghai Road 51, Liaoning, 121001 China

**Keywords:** Intracerebral haemorrhage, Endoscopic evacuation, Stereotactic aspiration, Craniotomy, Randomised controlled trial

## Abstract

**Background:**

Hypertensive intracerebral haemorrhage (HICH) is the most common form of haemorrhagic stroke with the highest morbidity and mortality of all stroke types. The choice of surgical or conservative treatment for patients with HICH remains controversial. In recent years, minimally invasive surgeries, such as endoscopic evacuation and stereotactic aspiration, have been attempted for haematoma removal and offer promise. However, research evidence on the benefits of endoscopic evacuation or stereotactic aspiration is still insufficient.

**Methods/design:**

A multicentre, randomised controlled trial will be conducted to compare the efficacy of endoscopic evacuation, stereotactic aspiration and craniotomy in the treatment of supratentorial HICH. About 1350 eligible patients from 10 neurosurgical centres will be randomly assigned to an endoscopic group, a stereotactic group and a craniotomy group at a 1:1:1 ratio. Randomisation is undertaken using a 24-h randomisation service accessed by telephone or the Internet. All patients will receive the corresponding surgery based on their grouping. They will be followed-up at 1, 3 and 6 months after surgery. The primary outcome is the modified Rankin Scale at 6-month follow-up. Secondary outcomes include: haematoma clearance rate; Glasgow Coma Scale 7 days after surgery; rebleeding rate; intracranial infection rate; hospitalisation time; mortality at 1 month and 3 months after surgery; the Barthel Index and the WHO quality of life at 3 months and 6 months after surgery.

**Discussion:**

The trial aims to investigate whether endoscopic evacuation and stereotactic aspiration could improve the outcome of supratentorial HICH compared with craniotomy. The trial will help to determine the best surgical method for the treatment of supratentorial HICH.

**Trial registration:**

ClinicalTrials.gov, ID: NCT02811614. Registered on 20 June 2016.

**Electronic supplementary material:**

The online version of this article (doi:10.1186/s13063-017-2041-1) contains supplementary material, which is available to authorized users.

## Background

Hypertensive intracerebral haemorrhage (HICH) is a crucially important neurological emergency characterised by high fatality and disability rates. HICH accounts for about 70% of all intracerebral haemorrhage (ICH) cases. It affects about four million people worldwide each year and the median mortality at 1 month is 40% [1, 2]. Many survivors remain severely disabled, and only 12% of the survivors could live independently with minor handicap after 6 months, posing a huge burden on society and families [3, 4].

Theoretically, surgery has the potential to improve neurological recovery after ICH since early removal of the haematoma might reduce nerve tissue damage, possibly by relieving local ischemia or the removal of noxious chemicals [5–7]. However, the effectiveness of surgery (mainly refers to craniotomy) in the treatment of ICH remains controversial [8]. Several prospective randomised controlled trials (RCTs) have been undertaken during the past four decades, but the results of most individual trials have failed to demonstrate improvement in outcome in surgically treated patients [9–15]. Results of the International Surgical Trial in Intracerebral Haemorrhage (STICH) trial indicate that patients with spontaneous, supratentorial ICH in neurosurgical units show no overall benefit from early surgery when compared with initial conservative treatment [15]. The STICH II trial, a study based on subgroup analysis of the STICH trial, confirms that early surgery does not increase the rate of death or disability at 6 months and might have a small but clinically significant survival advantage for patients with spontaneous, superficial ICH [16].

Minimally invasive surgery generally refers to the concept of creating minimal trauma to normal brain tissue during the process of removing haematoma. This stands in distinction to open craniotomy in which a large bone flap is created, the brain is exposed, retracted and manipulated to inspect the site of bleeding, and blood is suctioned from multiple areas [5]. Two main types of minimally invasive surgery have been attempted for haematoma removal: endoscopic evacuation and stereotactic aspiration. In endoscopic evacuation, a small burr hole is created and an endoscope is inserted through normal brain tissue into the haematoma with the help of an introducer. Suction and irrigation are applied to remove the haematoma. Auer reported the first randomised trial of endoscopic-guided haemorrhage evacuation with a sample size of 100 [9]. Results indicated that endoscopic surgical evacuation offered promise as a means to maximise haematoma evacuation while minimising damage to normal tissue. Stereotactic aspiration involves using image guidance to place a catheter into the main body of the haematoma and aspirating blood. A catheter is left in the body of the haematoma, and during the course of several days, repeated small doses of thrombolytic agents are instilled via the catheter into the brain to clear the left haematoma slowly. Recently, several studies exploring the efficacy of minimally invasive surgery compared with craniotomy or medical treatment have been carried out, but none of them provided sufficient evidence regarding the choice of treatment [17–20]. Until now, no results of large-scale RCTs comparing the efficacy of endoscopic evacuation, stereotactic aspiration and craniotomy in patients with ICH have been reported. Here, we designed a multicentre, RCT to compare and verify the safety and efficacy of endoscopic evacuation and stereotactic aspiration.

## Methods/design

### Objectives

This trial primarily aims to investigate whether, and to what extent, endoscopic evacuation and stereotactic aspiration could improve the outcome of supratentorial HICH compared with craniotomy. This trial will also help to better define the indications for different surgical methods and help to determine the best surgical method for the treatment of supratentorial HICH. Another objective is to evaluate the separate effects of different surgical treatments on patients’ independency, activities of daily living, and quality of life.

### Study design

**Fig. 1:**
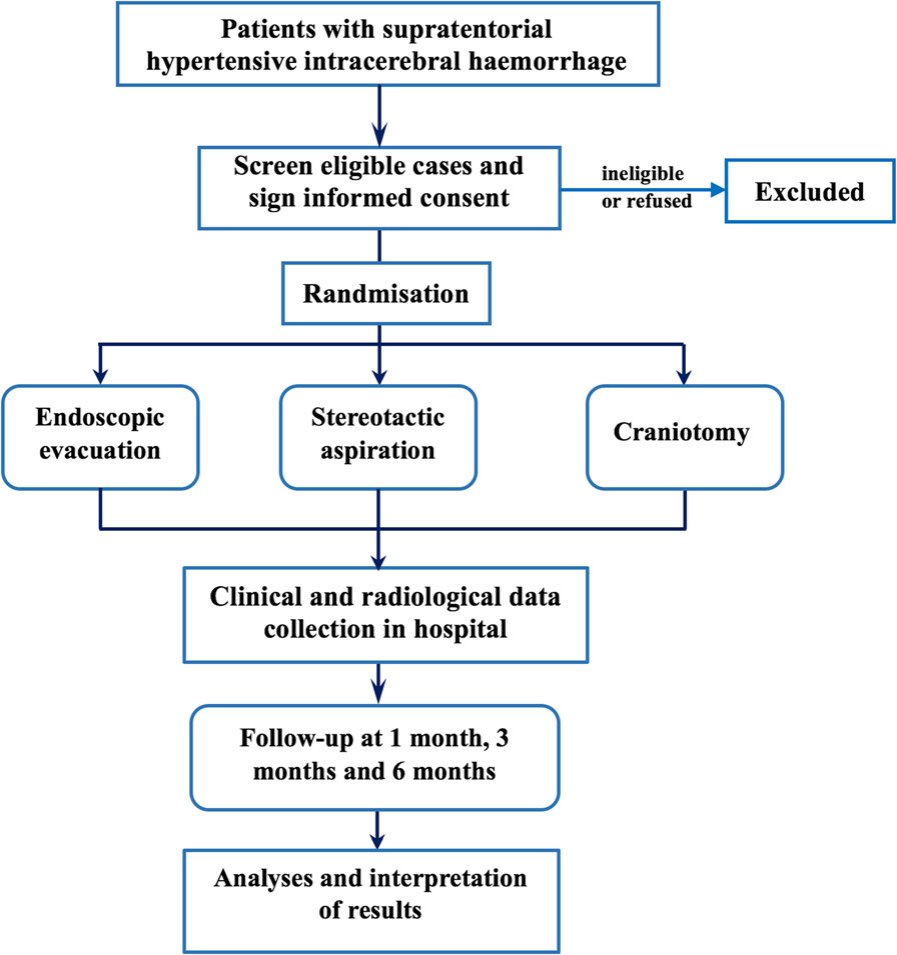
Flowchart of the study design

**Fig. 2:**
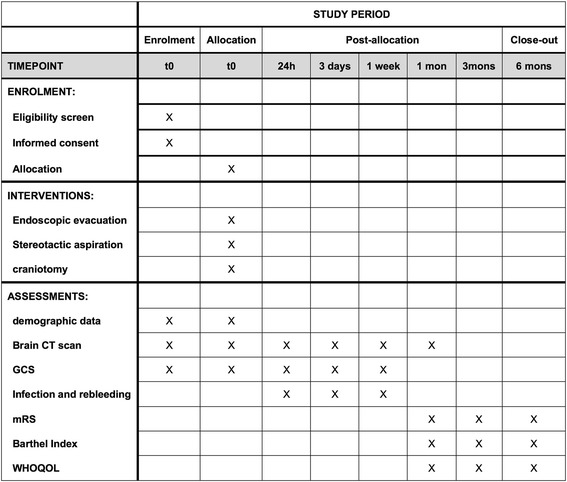
Standard Protocol Items: Recommendations for Interventional Trials (SPIRIT) figure: the schedule of enrolment, interventions and assessments. Abbreviations: *h* hour, *mo* month, *mons* months, *GCS* Glasgow Coma Scale, *mRS* modified Rankin Scale, *WHOQOL* the World Health Organisation Quality of Life questionnaire

This is a multicentre, randomised controlled, open-label, sequentially designed nonprofit study (ClinicalTrials.gov ID: NCT02811614) involving 10 neurosurgical centres. Patients with newly diagnosed supratentorial HICH according to our inclusion criteria will be enrolled in this study. A follow-up period of 6 months will be sufficient to show the outcome and prognosis of surgical treatment. A flowchart of this study is shown in Fig. 1 and the Standard Protocol Items: Recommendations for Interventional Trials 2013 (SPIRIT) Checklist is presented as Additional file 1. The SPIRIT schedule of enrolment, interventions and assessment is given in Fig. 2.

The trial will be conducted in the departments of neurosurgery at 10 Chinese hospitals: (1) The Chinese PLA General Hospital, Beijing, (2) The First Affiliated Hospital, Sun Yat-sen University, Guangzhou, (3) The First Hospital of Jilin University, Changchun, (4) The Second Affiliated Hospital of Zhejiang University School of Medicine, Hangzhou, (5) The Second Hospital of Jilin University, Changchun, (6) Tianjin Huanhu Hospital, Tianjin, (7) Jinzhou Central Hospital, Jinzhou, (8) Jingzhou Central Hospital, Jingzhou, (9) Dalian Central Hospital, Dalian and (10) Wuhan Integrated TCM and Western Medicine Hospital, Wuhan. All surgeons taking part in the trial have been trained by an annually held national continuing education program by the Chinese PLA General Hospital on endoscopic evacuation and stereotactic aspiration. These three surgical techniques have been routinely used in all centres prior to the start of the trial. Patients who refuse surgery and patients from the Department of Neurology with matched basic clinical characteristics will be assigned to a group for future statistical analysis.

### Eligibility criteria

#### Inclusion criteria

1. Men and women aged 18–75 years old
2. Presenting with supratentorial HICH confirmed by brain computed tomography (CT) scan, haematoma volume ≥30 mL
3. Glasgow Coma Scale (GCS) score ≥5
4. Patients are admitted to the above-mentioned hospitals within 24 h of ictus
5. Patients or their dependents (when patients are in coma) provide written consent

#### Exclusion criteria

1. The haemorrhage is caused by a cerebral tumor, coagulopathy, aneurysm or arteriovenous malformation
2. If there is concurrent head injury or history of head injury
3. If there are multiple hemorrhagic lesions
4. Patients with severe dementia or disability
5. Patients who already have indications of terminal brain herniation
6. Patients with concomitant diseases that will affect their life expectancy
7. Main body of the haematoma is located in the ventricular system
8. Pregnant women

### Sample size and randomisation

#### Sample size

It has been reported that the rate of poor outcome of patients receiving craniotomy with a supratentorial haematoma volume >30 mL was 49 to 67% and minimally invasive surgery might decrease the rate of poor outcome by about 8 to 22% compared with craniotomy [21–23]. Our recent retrospective study showed that endoscopic surgery lowered the risk of poor outcome in patients with supratentorial HICH by nearly 28% in comparison with craniotomy [24]. We assume that the neuroendoscopic surgery and stereotactic aspiration could reduce the risk of poor outcome from 58 to 49%. A sample size of 1230 would be required to show a 9% benefit from minimally invasive surgery (two-way *P* < 0.05) with 80% power. A sample size of 1350 (450 for each group) was chosen to allow for some loss to follow-up and a small crossover rate.

#### Randomisation

To minimise selection bias and accidental bias, patients will be randomised to an endoscopic evacuation group, a stereotactic aspiration group or a craniotomy group by means of central simple randomisation after the informed consent has been signed. Neither the neurosurgeons nor the patients could know the grouping beforehand. Randomisation will be performed using a web-based 24-h randomisation system. Randomisation must take place within 24 h of ictus and patients will be operated on within 12 h of randomisation. During the randomisation process the neurosurgeon is informed of the treatment group the patient is allocated to and should record this. Best medical treatment must begin as soon as possible and continue throughout follow-up in all patients.

### Surgical procedures

#### Endoscopic evacuation

In this intervention group, patients will receive endoscopic surgery under general anesthesia. A 3-cm skin incision is made according to the position of the haematoma on brain CT scan. The approaches include the middle frontal gyrus approach for anterior basal ganglia haemorrhages that are not elongated but rather are more spherical; a parietooccipital burr hole is created to treat posterior basal ganglia and thalamic haemorrhages. In cases involving superficial lobar haemorrhages, a burr hole is used directly at the location where the lesion comes closest to the surface [25]. Then, a bone flap of about 2 cm in diameter is made and the dura mater incised in a cruciate fashion. Using a newly developed endoscopic introducer (China National Invention Patent: 201210066281.1) an appropriate working channel for the endoscope is made. First, the blunt puncturing lever is used to make an entry incision impaled in a predetermined position and then the inner core is removed. Second, syringe suction is used to determine whether the puncture lever has been positioned in the right place. After confirmation, the transparent cannula is placed outside along the puncture lever. Through the space made by the transparent introducer, we can carefully evacuate the intracerebral haematoma under endoscopic surveillance. Plasminogen activator (urokinase) will not be injected after endoscopic surgery.

#### Stereotactic aspiration

This group of patients will receive haematoma evacuation by stereotactic aspiration under local anesthesia and intravenous sedation. First, the patient will undergo a brain CT scan with slice thickness less than 5 mm and the CT imaging data in Digital Imaging and Communications in Medicine (DICOM) format will be collected. Then, the neurosurgeon will make a three-dimensional reconstructed image of haematoma using 3D Slicer version 4.5 software (http://www.slicer.org/; Harvard University, Boston, MA, USA) and measure the puncture depth from the puncture site to the target point. The target point is chosen on the CT scan with the largest expansion of the hematoma and special attention is paid to stay away from important cortical function areas. The puncture site is measured and marked on the head, then a puncture needle with a catheter is inserted to the premeasured depth. The hematoma will be withdrawn gently using a syringe (diluted with saline if the blood is thickened) until no more blood can be withdrawn. Urokinase will be injected through the catheter into the haematoma to dissolve the residual haematoma afterward. Three thousand to six thousand units urokinase will be injected into the haematoma two or three times daily for 2–4 days. A CT scan should be performed 24 and 72 h after surgery. The drilling catheter will be removed when more than 80% of the original haematoma volume has been removed or no more haematoma can be aspirated.

#### Craniotomy

Patients in this group will receive haematoma evacuation by craniotomy under general anesthesia. Well-trained neurosurgeons will decide the surgical approach according to haematoma location and size on CT scan. To minimise the damage to normal brain tissue, the operative incision should be made as small as possible. After removing the bone and incising the dura mater, the cortex is incised to reach the haematoma on the basis of protecting the functional cortical areas and blood vessels. The haematoma will be evacuated as much as possible with the help of an operative microscope. After careful hemostasis, the blood pressure should be moderately elevated to confirm that there has been no bleeding site. If the brain tissue swells significantly after haematoma evacuation, then the bone flap should be abandoned. A drainage catheter is left in the haematoma cavity and will be pulled out at 24–48 h after surgery. Urokinase will not be injected afterward.

#### Concomitant care and interventions

All patients will be cared for in a neurosurgical intensive care unit (NICU) until they are considered stable enough to move to an intermediate care or general unit. Neurological status will be monitored in the NICU using the GCS and hourly neurological evaluation which includes vital signs, level of consciousness and limb muscular strength.

### Outcome measurements

The primary outcome of this study is the degree of disability estimated using the modified Rankin Scale (mRS) 6 months after ictus. A poor outcome is defined as death or dependency with a mRS of 3–6. Living patients’ independence in activities of daily lives and quality of life will be a highlight of this study. We will also measure patients’ quality of life and their performance in activities of daily living using the Barthel Index and the World Health Organisation (WHO) Quality of Life-BREF at 3- and 6-month follow-ups. Other clinical information that will also be measured include: haematoma clearance rate evaluated at 72 h after surgery, operation time, intraoperative blood loss, postoperative GCS score 7 days after surgery, rebleeding rate, days of NICU stay, intracranial infection rate during hospitalisation, 1-month mortality, 3-month mortality, and mRS scores at 3 months.

### Data collection and follow-up

A Case report Form (CRF) with detailed record rules has been developed in order to ensure accurate data collection. Patients’ personal data are made anonymous and numbered by the system. A list of patient names and study numbers is kept in a separate file to ensure that patients’ confidentiality is maintained. Demographic, clinical and neurological data are recorded at the time of enrolment and throughout follow-ups. The filled CRFs should be sent to the trial secretary expeditiously.

**Fig. 3:**
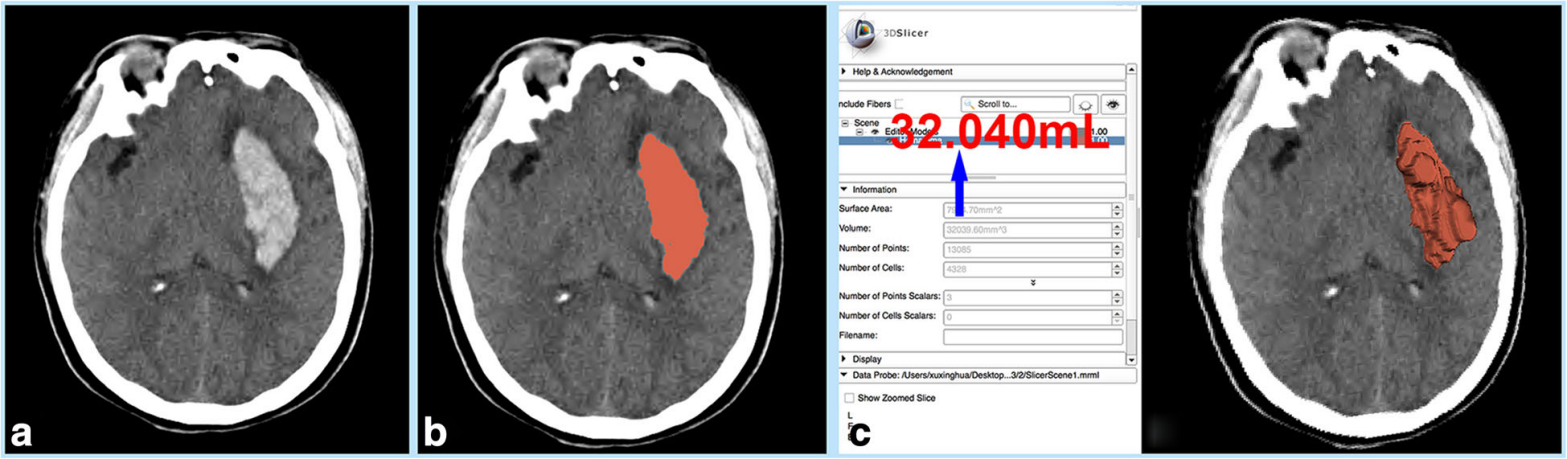
Haematoma volume measurement using the software 3D Slicer. **a** Preoperative brain computed tomography (CT) scan confirming left external capsular haemorrhage. **b** Automatic depiction of the haematoma using the threshold effect. **c** 3D reconstruction and volume measurement of the haematoma

Copies of the CT scans at enrolment, 1 day, 3 days and 7 days after surgery will be sent to the trial secretary together with the CRFs. The preferred method of sending a CT scan is in DICOM-compatible format. DICOM images are sent anonymously with only the patient identifier visible. The images will be analysed by trained readers blinded to treatment group and patient identifier. In past studies, almost all haematoma volumes have been calculated by the ABC/2 formula. However, there is significant estimation error using the ABC/2 formula to calculate haematoma volume, especially in large, irregular haematomas [26]. In this trial, we will use a precise and free method, the software 3D Slicer, to measure both preoperative and postoperative haematoma volumes (Fig. 3).

Follow-up data will be collected at 1 month, 3 months and 6 months after onset. All patients are encouraged to make a regular return visit to the hospital. Patients who cannot make a regular return visit will be followed-up through telephone or postal questionnaires. Our goal is to achieve more than 95% follow-up by means of full cooperation of all the investigators.

### Statistical analysis

Statistical analysis will be on a “intention-to-treat” basis. Measurement data will be described using mean and standard deviation or median and interquartile range, while count data will be described using indexes such as percentage, ratio and relative risk. The primary analysis will be a simple categorical frequency comparison using the chi-squared test for prognosis based on favourable and unfavourable outcomes at 6 months. The continuous variables will be analysed by one-way analysis of variance (ANOVA). Logistic regression analysis will be undertaken to adjust for covariates. Subgroup analyses include age, haematoma volume, haematoma location, time from ictus to surgery, GCS at hospitalisation, different centres, and the presence of an intraventricular haemorrhage, etc. All statistical analyses will be performed using SPSS statistics 22 (IBM Corp., Armonk, NY, USA). A value of two-side *P* < 0.05 was considered statistically significant.

### Data monitoring and quality control

The Data Monitoring Committee considers data from interim analyses and reports to the Trial Steering Committee. Interim analyses are strictly confidential and the committee will only recommend stopping the trial early if one or other treatment shows an advantage at a very high significance level. The coordinating centre (Chinese PLA General Hospital, Beijing) is responsible for maintaining computerised databases containing all data related to the trial, the quality of computerised information, conducting statistical analyses, preparing reports for the Data Monitoring Committee, and all correspondence in relation to the trial. To better control the quality of this study, all neurosurgeons performing the operation will be given a short-term centralism training before the trial begins.

## Discussion

ICH has always been a controversial topic in neurology and neurosurgery. Endoscopic evacuation and stereotactic aspiration have been investigated by comparison with conservative treatment or traditional craniotomy [9, 20, 27–31]. However, since most studies are nonrandomised and retrospective, the advantage of minimally invasive surgery remains unproven. Furthermore, the optimal timing of haematoma evacuation and the optimal time to maximum haematoma removal remains unclear. Endoscopic evacuation is a rapid process of haematoma volume reduction while stereotactic aspiration is a gradual process of haematoma volume reduction. It is still very difficult to select individualised treatment for each patient. By subgroup analyses of our large-scale multicentre RCT, we will try to find out the answers to these questions. Besides, with the improvement of living standards and the development of medical science, patients’ quality of life is attracting more and more attention. In this study we will make a study of quality of life in surviving HICH patients and investigate the factors that influence quality of life. Although it is controversial, surgical treatment still plays an important role in the management of HICH. Exploring a more efficient surgical method in order to improve clinical outcome and prognosis of HICH is essential and urgent. We hope that our findings may be of benefit to HICH patients and will provide further evidence for neurosurgeons and HICH patients when choosing surgical options.

### Trial status

The trial was first designed in July 2016 and subject recruitment began in November 2016.

## Additional file

Additional file 1: SPIRIT Checklist. (PDF 83 kb)

## Abbreviations

3D: Three-dimensional
ANOVA: Analysis of variance
BI: Barthel Index
CRF: Case Report Form
CT: Computed tomography
DICOM: Digital Imaging and Communications in Medicine
GCS: Glasgow Coma Scale
HICH: Hypertensive intracerebral haemorrhage
IBM: International Business Machine
ICH: Intracerebral haemorrhage
IRB: Institutional Review Board
mRS: Modified Rankin Scale
NICU: Neurosurgical intensive care unit
PLA: People’s Liberation Army
SPSS: Statistic Package for Social Science
STICH: International surgical trial in intracerebral haemorrhage
WHO: World Health Organisation
